# Asymmetry of price returns—Analysis and perspectives from a non-extensive statistical physics point of view

**DOI:** 10.1371/journal.pone.0188541

**Published:** 2017-11-30

**Authors:** Łukasz Bil, Dariusz Grech, Magdalena Zienowicz

**Affiliations:** 1 Faculty of Physics, University of Warsaw, 5 Pasteur Str., PL-02-093 Warsaw, Poland; 2 Institute of Theoretical Physics, University of Wrocław, 9 Max Born Sq., PL-50-204 Wrocław, Poland; 3 Faculty of Environmental Engineering and Geodesy, Wrocław University of Environmental and Life Sciences, 55 Grunwaldzka Str., PL-50-357 Wrocław, Poland; Universidad Veracruzana, MEXICO

## Abstract

We study how the approach grounded on non-extensive statistical physics can be applied to describe and distinguish different stages of the stock and money market development. A particular attention is given to asymmetric behavior of fat tailed distributions of positive and negative returns. A new method to measure this asymmetry is proposed. It is based on the value of the non-extensive Tsallis parameter *q*. The new quantifier of the relative asymmetry level between tails in terms of the Tsallis parameters *q*^±^ is provided to analyze the effect of memory in data caused by nonlinear autocorrelations. The presented analysis takes into account data of separate stocks from the main developing stock market in Europe, i.e., the Warsaw Stock Exchange (WSE) in Poland and—for comparison—data from the most mature money market (Forex). It is argued that the proposed new quantifier is able to describe the stage of market development and its robustness to speculation. The main strength is put on a description and interpretation of the asymmetry between statistical properties of positive and negative returns for various stocks and for diversified time-lags Δ*t* of data counting. The particular caution in this context is addressed to the difference between intraday and interday returns. Our search is extended to study memory effects and their dependence on the quotation frequency for similar large companies—owners of food-industrial retail supermarkets acting on both Polish and European markets (Eurocash, Jeronimo-Martins, Carrefour, Tesco)—but traded on various European stock markets of diversified economical maturity (respectively in Warsaw, Lisbon, Paris and London). The latter analysis seems to indicate quantitatively that stocks from the same economic sector traded on different markets within European Union (EU) may be a target of diversified level of speculations involved in trading independently on the true economic situation of the company. Our work thus gives indications that the statement:” where you are is more important than who you are” is true on trading markets.

## Introduction

The behavior of financial markets due to rich, complex and intriguing dynamics of stock and financial trading (see, e.g., [1]–[15]) has been in focus of interest of physicists for a quite long time. The presence of fat tailed distributions of returns and their power law features [16]–[22] seem to be crucial among variety of problems involved in complexity of this trading. The fat-tailed distributions indicate that one is allowed to earn (or loose) much more than well known century-old Gaussian model is able to predict [23]. The latter one simply underestimates the probability of large events and therefore cannot be used as an adequate model in risk analysis. Events such as the 1987 stock market crash where the Standard & Poor 500 index—the leading on US stock market—dropped by a magnitude of over 20 standard deviations, or many other crashes observed around the world in long stock market history, would have never happened if the probability distribution of financial returns was given by the Gaussian law.

The most relevant quantitative characteristics of the financial dynamics is therefore hidden in the functional form of the return distribution. The nature of fat tails is not known so far in details. It is agreed however, that their source must lie in memory effects in data beyond the non-vanishing simple two point autocorrelation function. It is well established fact that nonlinear correlations between returns generate fat tails of their probability distribution (see, e.g, [15, 24, 25]). Such nonlinear correlations are visible for instance as volatility clustering, i.e., volatility correlations which are observed in the simplest case as autocorrelations between absolute returns. Also multifractal properties of data in time series are likely to produce similar results [26]. Thus the quantitative description of shape of distribution tails of financial data may serve as important global quantifier which identifies much more complex nonlinear phenomena existing underneath. Such phenomena are usually difficult for simultaneous detection, proper identification and description in complex systems of any kind. Therefore it is interesting to explore the local (in time) statistical properties of a complex stock system and its evolution. They reflect much more diversified internal organization of financial complexity.

The well-identified fact connected with fat tails of return distribution is the so-called inverse cubic power law [27]–[29], which has been shown to hold for different stock market indices [22], [27]–[31] across different sizes of stocks, in different time periods and for time-lags in returns ranging from seconds to even one month. Its accuracy has been reported over as many as 80 standard deviations for some stock markets. It can be applied to commodity market as well as to traded currency exchange rates (see, e.g., [32]–[34]).

Let us briefly recall this law together with notation used in this paper. One usually considers returns *R*(*t*, Δ*t*) calculated for the chosen time-lag Δ*t* for the time series of prices *p*(*t*), where *t* = 1, 2, …, *T* numerates data in discrete time window, as follows:

$$\begin{array}{c} R\left(t,\Delta t\right)=\frac{p(t)-p(t-\Delta t)}{p(t-\Delta t)} \end{array}$$ (1)

Often the so called logarithmic return is defined *R*^*L*^(*t*, Δ*t*) = ln *p*(*t*) − ln *p*(*t* − Δ*t*) which is equivalent to *R*(*t*, Δ*t*) if |*R*(*t*, Δ*t*)| ≪ 1. We will use further within this paper the standard definition of Eq (1), however all the following results were checked by us to remain quantitatively the same also for logarithmic returns.

In order to compare statistically different stocks the normalization procedure should be performed. Therefore the normalized returns *r*(*t*, Δ*t*) have to be introduced:

$$\begin{array}{c} r\left(t,\Delta t\right)=\frac{R\left(t,\Delta t\right)-{\left\langle R\left(t,\Delta t\right)\right\rangle }_{T}}{\sigma_{T}} \end{array}$$ (2)

where 〈*R*(*t*, Δ*t*)〉_*T*_ means the mean value of *R* over the time window of length *T*, while *σ*_*T*_ stands for the standard deviation $\sigma_{T}^{2}\equiv {\langle R^{2}\rangle }_{T}-{\langle R\rangle }_{T}^{2}$.

The inverse cubic law states that the cumulated probability *P*(|*r*|>*x*) for the given time interval (time-lag) Δ*t* has the power law form:

$$\begin{array}{c} P\left(|r|>x\right)∼x^{-\zeta } \end{array}$$ (3)

with particular exponent value *ζ* ≈ 3. This value of scaling exponent is claimed almost independent on Δ*t* (see [28], [29]), size of stock and the period of measurement.

A very nice formalism which explains the origin of power laws of probability distributions may be grounded on statistical physics and generalization of Boltzmann-Gibbs entropy. This generalized non-extensive entropy *S*_*q*_ proposed by Tsallis [35], [36] follows the scaling properties of multifractals [37] and reads:

$$\begin{array}{c} S_{q}=k\frac{1}{q-1}\left(1-\int \rho {\left(x,t\right)}^{q}dx\right) \end{array}$$ (4)

The main ingredients of definition in Eq (4) are: the probability density function *ρ*(*x*, *t*) of the stochastic variable *x* (generally time dependent), the single continuous arbitrary real parameter *q* and the Boltzmann constant *k*. The Tsallis non-extensive entropy is reduced in the limit *q* → 1 to the classical extensive Boltzmann-Gibbs form

$$\begin{array}{c} S_{BG}=-k\int \rho \left(x,t\right)\ln\rho \left(x,t\right)dx \end{array}$$ (5)

Hence, the non-extensive entropy *S*_*q*_ is a *generalization* of *S*_*BG*_, and makes *not an alternative version* of *S*_*BG*_.

It is well known that an extensive statistical system obtains the maximum of *S*_*BG*_ entropy if the probability density function of its states *ρ*(*x*) is gaussian. Similarly, the maximum of Tsallis *q*-entropy yields another equilibrium distributions but of *power law* type instead of *exponential* ones. Indeed, one can show that the optimization of *S*_*q*_ yields, for the stationary state, the following distribution of probabilities called *q*-normal or Tsallis distribution [36]:

$$\begin{array}{c} P\left(x\right)=N_{q}{\left(1+B_{q}x^{2}\left(q-1\right)\right)}^{\frac{1}{\left(1-q\right)}} \end{array}$$ (6)

where

$$\begin{array}{c} B_{q}={\left[\left(5-3q\right)\sigma^{2}\right]}^{-1} \end{array}$$ (7)

and *σ*^2^ is the standard variance of the sample. The normalization constant *N*_*q*_ for 1 < *q* < 3 (this case is most interesting because the power law form of distribution tails is developed for large |*x*|) is expressed by Gamma-Euler function Γ as

$$\begin{array}{c} N_{q}=\frac{\Gamma (\frac{1}{q-1})}{\Gamma (\frac{3-q}{2q-2})}\sqrt{\frac{q-1}{\pi }B_{q}} \end{array}$$ (8)

Note, that Eq (6) takes the power law form *P*(*x*) ∼ *x*^2/(1 − *q*)^ for |*x*|→∞. At the level of cumulative probability distribution one arrives then with:

$$\begin{array}{c} P\left(|r|>x\right)∼x^{-\frac{3-q}{q-1}} \end{array}$$ (9)

Formally Eq (9) coincides with Eq (3) and recovers the inverse cubic law for *q* = 3/2. However, the non-extensive approach is capable to go much further—beyond the inverse cubic law and the Gaussian law—since it continuously passes through all intermediate cases of fat tailed distributions in a compact and very economic way. This can be done just by altering the value of *q* parameter. Hence, an open question in non-extensive approach is often raised about the meaning, value and behavior of the Tsallis parameter *q* beyond the *q* = 1 case corresponding to independent *x* variables. When moved into finance, this problem seems to be even more interesting and intriguing. A comparison of corresponding properties between different markets and detailed analysis of individual stocks may be the way to get at least partial answer to such question. It will also be of great advantage for investors and traders on the stock market.

Thus instead of searching for details of tails of some probability distribution (with precisely unknown functional form for the whole range of data) we will search for probability distribution well fitted to all parts of data—including the central and the edge part of empirical returns. Then we will determine the power law form of tails in terms of the main parameter describing the statistical properties of all data—not only fat tails. For the reasons clarified above the use of Tsallis distribution is supported in such approach by the expected link between microstructure of stock market (treated as the complex system) and macroscopic properties, i.e., observable data.

Hence, the *q*-normal distribution will be in focus of our interest in the next section while making a fit to real stock data and to most liquid money market data. However, more precisely, the main strength of this article will be put on the *asymmetry* between statistical properties of positive and negative returns discussed and interpreted in terms of Tsallis statistics. This will be done for various stocks and for diversified time lags Δ*t* of data counting (see Eqs (1) and (2)). The particular caution will be addressed in this context to difference between intraday and interday returns. In the final section 3 we collect remarks and conclusions drawn from the real data analysis with hope to indicate possible practical meaning and use of Tsallis parameter in stock market analysis.

## Analysis of stock and money market data

Let us begin from the analysis of data from the biggest developing stock market in Europe, i.e., the Warsaw Stock Exchange (WSE) in Poland. For this purpose we examined the normalized and centered (according to Eq (2)) returns of all 30 separate stocks that make up the content of the main WIG 30 stock index on this market. The statistics of these returns was found from the historic two years intraday and interday quotations taken from http://www.gpwinfostrefa.pl/; http://www.inwestoronline.pl/ in the period March 27, 2013—March 31, 2015. For intraday quotations mid-prices data were used. All data have been initially checked in case of artificial breaks (empty stock quotes). Such breaks, if present, have been removed as they may introduce obvious artifacts in statistical analysis. The examined period counted finally *T* ∼ (7.6 ÷ 18) × 10^4^ data points (depending on particular stock) for the time-lag Δ*t* = 1 min.

These data were then used by us to construct the normalized and centered returns *r*(*t*) for other considered time-lags Δ*t*—separately for each company. In the case of intraday returns (Δ*t* = 5 ÷ 60 min) they were collected with comparable statistics as for Δ*t* = 1 min. The interday returns for Δ*t* = 1 ÷ 4 days have been calculated with slightly smaller statistics of (5 ÷ 13) × 10^4^ points but of the same order as for intraday returns. The final results for various companies turned out to be qualitatively identical and quantitatively very close to each other. Therefore, we present them only for chosen companies from WIG 30 in this paper. The chosen stocks are good representative examples of different economic sectors in Poland like: banking (PKO BP), telecommunication (Orange), fuel and energy sector (PKN Orlen) or insurance sector (PZU). Our findings have been compared with the corresponding statistical properties of returns from capital weighted stock index WIG 30. The latter one seems to be a good economic “reference frame” since it accommodates full variety of stocks. Therefore the whole stock index is more resistant to speculation of investors who may speculate easier within just one sector or with stocks of one company.

**Fig 1:**
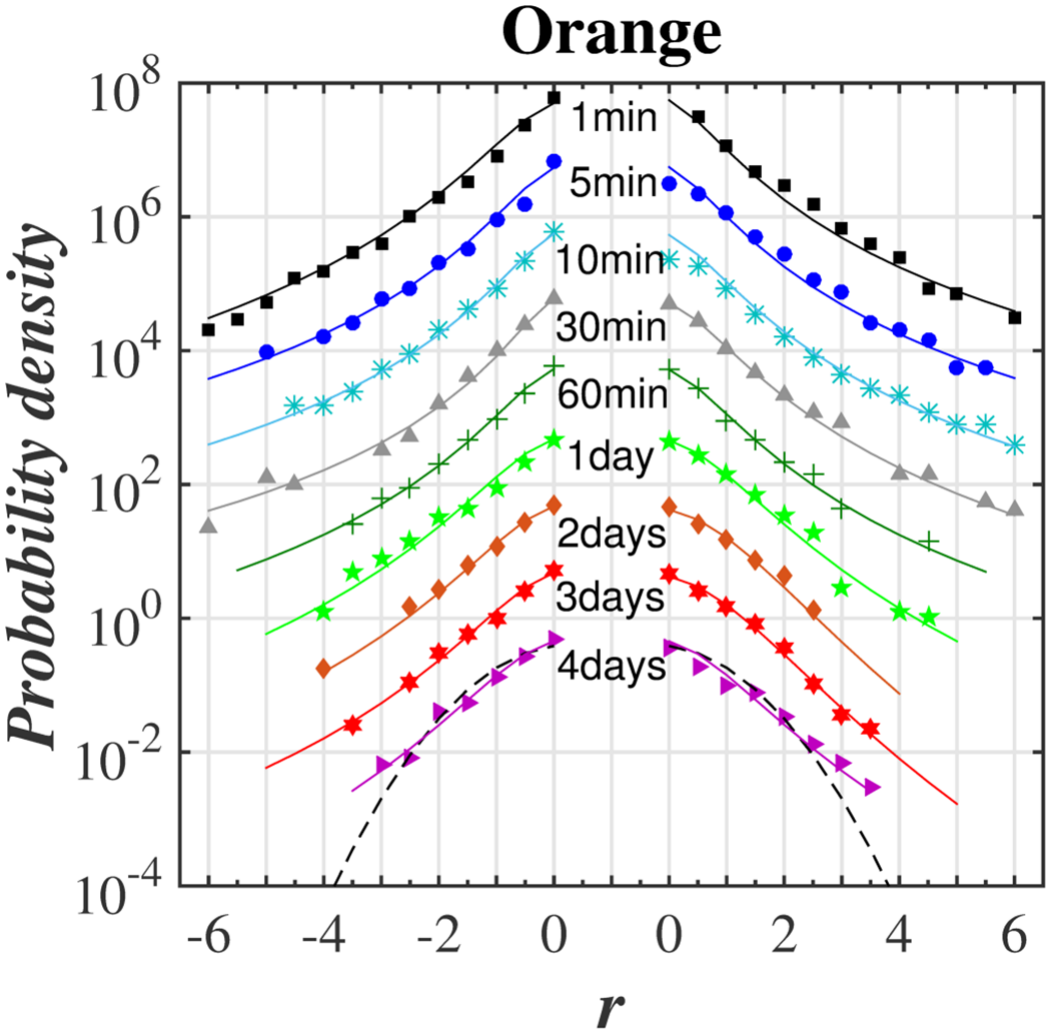
Probability density function in log-linear scale of normalized and centered returns for Orange stock from WIG 30 stock index, calculated for various time-lags Δ*t* in the period: March 2013—March 2015. The plots for different Δ*t* were drawn as dots and are relatively vertically shifted for better display. Dashed line corresponds to Gaussian distribution while solid lines present the best fit of *q*-normal Tsallis distribution of positive and negative returns separately.

**Fig 2:**
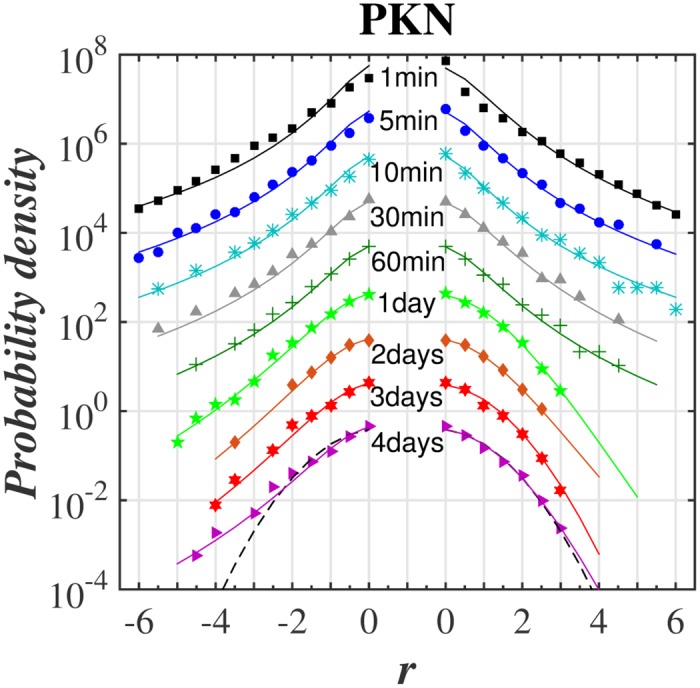
Same as in Fig 1 for PKN orlen stock.

**Fig 3:**
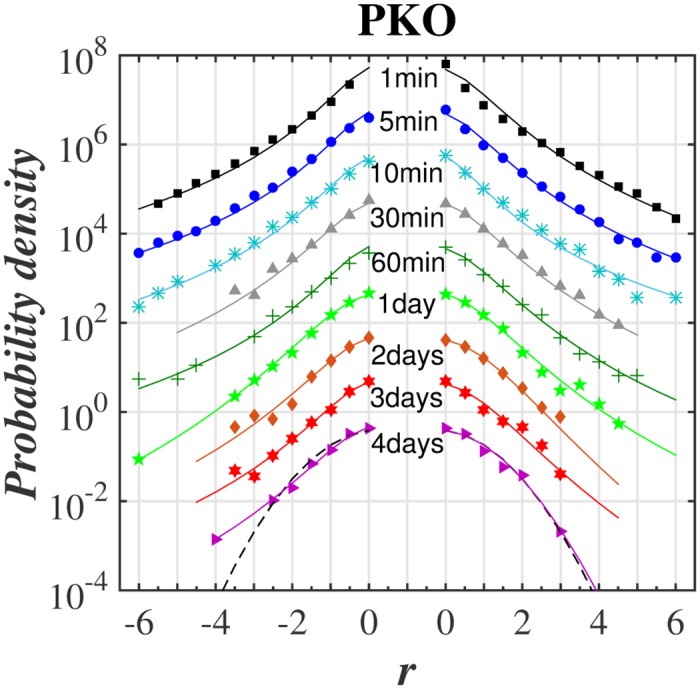
Same as in Fig 1 for PKO BP stock.

**Fig 4:**
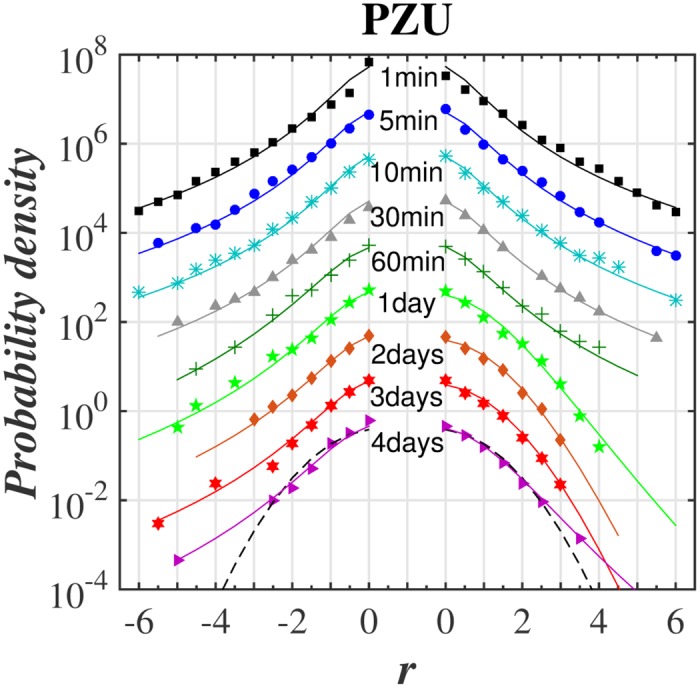
Same as in Fig 1 for PZU stock.

**Fig 5:**
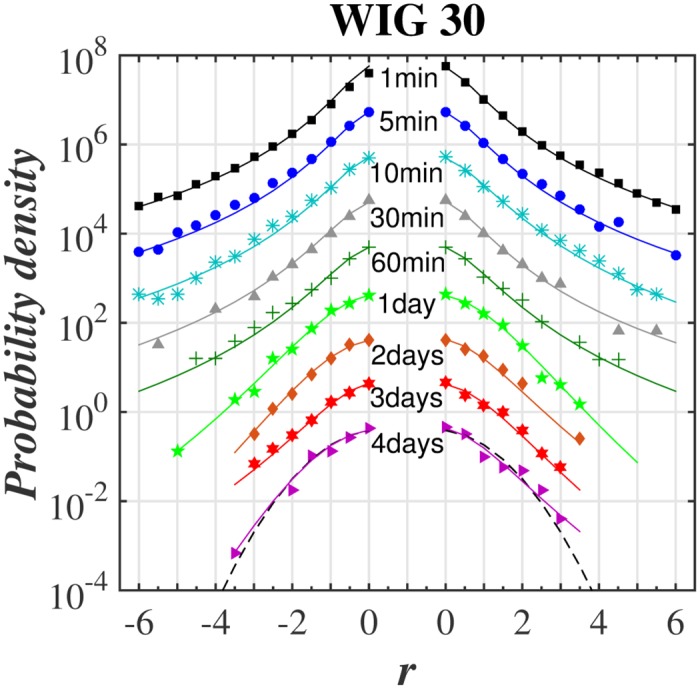
Same as in Fig 1 for the capital weighted WIG 30 stock index of Warsaw Stock Exchange (WSE).

**Fig 6:**
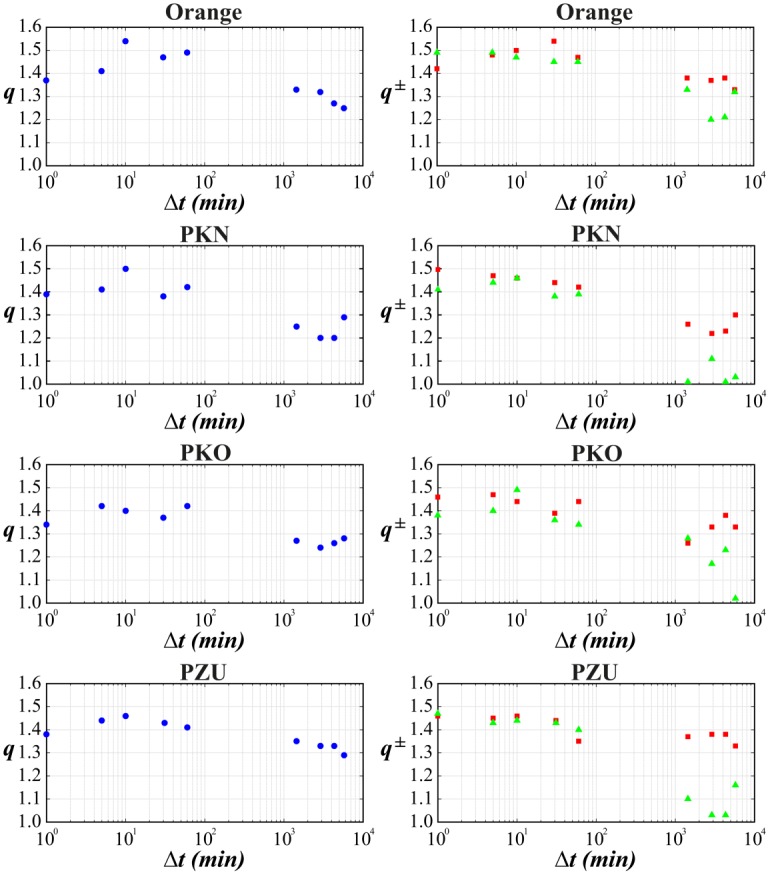
Dependence between Tsallis parameters. *q* (left panels) (blue point—all returns) and *q*^±^ (right panels) (green triangle—positive returns, red square—negative returns) found from Figs 1–4 versus the time-lag used for calculation of returns.

**Fig 7:**
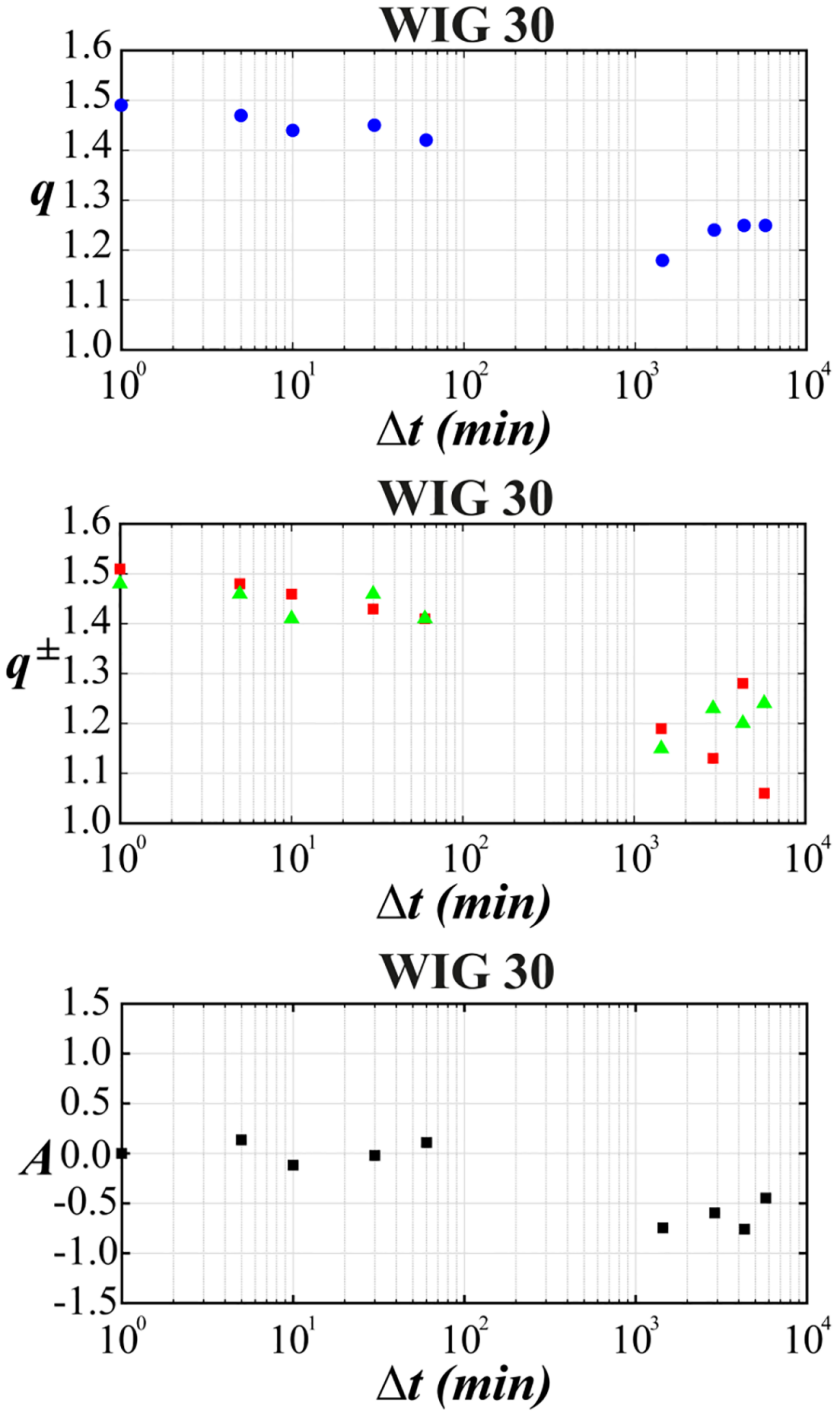
Same as in Fig 6 but for WIG 30 signal. The top panel indicates dependence of *q* on the time-lag for all returns (blue points). The middle panel shows dependence of *q*^±^ on the time-lag (green triangle—positive returns, red square—negative returns). The bottom panel shows dependence between skewness and the time-lag.

The main goal was to make a *q*-normal fit to distribution of returns for various time-lags. The quality of such fit for selected stocks from WIG 30 index and for the variety of time-lags ranging from 1 min to 4 days is illustrated in series of plots in log-linear scale in Figs 1–4. Fig 5 shows additionally the interesting comparison with results for the whole WIG 30 stock index. We used the mean squared displacement method (MSD) to find in log-linear scale the best fitted profile of *q*-normal distribution. In all cases we fixed *B*_*q*_ via Eq (7) by estimating first the sample variance. Then only one remaining free parameter *q* of Tsallis distribution was fitted according to Eq (6). The fit was done twice—first for all returns what gives the best fit *q* value for all returns independently on their sign. Then the separate fits were performed for positive and negative returns independently. The latter fits are actually shown in Figs 1–5. They provide two different *q*^−^ and *q*^+^ values of Tsallis index respectively for the left and right tail of distribution. While making the fit according to Eq (6) we scanned the regime of *q*’s with the constant step Δ*q* = 2 × 10^−3^. Then the best fit result was rounded to two decimal places. Thus any difference in the second decimal place for *q*^±^ indicates already the actual difference between the fitted values *q*^+^ and *q*^−^. The results of fit to *q*-normal distributions (symmetric and asymmetric cases) are summarized in Table 1. For better visualization the Tsallis index has also been shown for separate stocks in Fig 6 as a function of time-lag Δ*t*. The corresponding plot for returns of the whole WIG 30 stock index was revealed in the top and the middle panels of Fig 7. We present in Table 2 the results of two samples Kolmogorov-Smirnov test (K-S) which aims to indicate if two samples (empirical and reconstructed from left (right) tail of *q*-normal distributions) have the same probability distribution. The corresponding *P* values of test are much larger than the significance level 0.05. Thus the null hypothesis is accepted that probability distributions of real return data have no significant difference comparing with *q*—normal distributions for given *q*^±^ from Table 1 with Δ*q*^±^ ≤ 0.005 bound on Tsallis parameter. This perfect agreement is seen also directly in plots of Figs 1–5.

**Table 1:** Results of *q*-normal distribution fit to statistics of returns for chosen stocks within WIG 30 index. Shown are also results of independent fit of Tsallis parameter *q*^±^ to the right (positive returns) and left (negative returns) tail of probability distribution.

Δ*t*	Tsallis index	OrangePL	PKN Orlen	PKO BP	PZU	WIG 30
1 min	*q*^−^	1.42	1.50	1.46	1.46	1.51
	*q*	1.45	1.45	1.41	1.46	1.49
	*q*^+^	1.49	1.41	1.38	1.47	1.48
5 min	*q*^−^	1.48	1.47	1.47	1.45	1.48
	*q*	1.48	1.44	1.43	1.44	1.47
	*q*^+^	1.49	1.44	1.40	1.43	1.46
10 min	*q*^−^	1.50	1.46	1.44	1.46	1.46
	*q*	1.50	1.46	1.46	1.46	1.44
	*q*^+^	1.47	1.46	1.49	1.44	1.41
30 min	*q*^−^	1.54	1.44	1.39	1.44	1.43
	*q*	1.47	1.38	1.37	1.43	1.45
	*q*^+^	1.45	1.38	1.36	1.43	1.46
60 min	*q*^−^	1.47	1.42	1.44	1.35	1.41
	*q*	1.47	1.42	1.42	1.40	1.41
	*q*^+^	1.45	1.39	1.34	1.40	1.41
1 day	*q*^−^	1.38	1.26	1.26	1.37	1.19
	*q*	1.35	1.25	1.27	1.35	1.18
	*q*^+^	1.33	1.07	1.28	1.10	1.15
2 days	*q*^−^	1.37	1.22	1.33	1.38	1.13
	*q*	1.32	1.20	1.24	1.33	1.20
	*q*^+^	1.20	1.11	1.17	1.03	1.23
3 days	*q*^−^	1.38	1.23	1.38	1.38	1.28
	*q*	1.27	1.20	1.26	1.33	1.25
	*q*^+^	1.21	1.01	1.23	1.03	1.20
4 days	*q*^−^	1.33	1.30	1.33	1.33	1.06
	*q*	1.25	1.29	1.28	1.29	1.20
	*q*^+^	1.16	1.03	1.02	1.16	1.24

**Table 2:** P-values of two samples Kolmogorov-Smirnov test comparing real data distribution with *q* normal distribution for individual stocks and the whole WIG 30 index (independent fit of left and right tail is performed).

Δ*t*	Orange		PKN		PKO		PZU		WIG 30	
	*q*^−^	*q*^+^	*q*^−^	*q*^+^	*q*^−^	*q*^+^	*q*^−^	*q*^+^	*q*^−^	*q*^+^
1 min	0.914	0.962	0.457	0.391	0.516	0.395	0.430	0.731	0.983	0.526
5 min	0.425	0.467	0.995	0.329	0.765	0.945	0.438	0.668	0.804	0.762
10 min	0.331	0.312	0.438	0.303	0.926	0.852	0.982	0.338	0.926	0.969
30 min	0.970	0.985	0.930	0.636	0.737	0.988	0.884	0.931	0.715	0.573
60 min	0.413	0.565	0.858	0.916	0.827	0.617	0.733	0.787	0.363	0.398
1 day	0.858	0.958	1,000	0.886	0.440	0.671	0.583	0.601	0.858	0.473
2 days	0.789	0.607	0.532	0.362	0.737	0.419	0.419	0.565	0.970	0.749
3 days	0.567	0.473	0.991	0.975	0.723	0.565	0.524	0.330	0.943	0.943
4 days	0.943	0.906	0.916	0.789	0.954	0.532	0.713	0.713	0.874	0.970

The first observation one can make from the quoted values of the main Tsallis index *q* (see Table 1 and Figs 6 and 7) is that the inverse cubic power law corresponding to *q* = 3/2 can be very well recognized for higher frequency quotations (Δ*t* = 1 min) of the whole WIG 30 index. Also the constituent stocks seem to obey this law for 1 < Δ*t* < 30 min however, usually with minor delay comparing with the main index, i.e., for slightly higher time-lags. Nevertheless, one observes a remarkable departure from the inverse cubic law already for time-lags Δ*t* ∼ 60 min. The Tsallis parameter reaches then *q* ≃ 1.4 corresponding to *ζ* = 4 (see Eq (3)). On the other hand, the value *q* = 1.2 suggested as asymptotic value for developed markets and obtained for such grown markets scarcely for Δ*t* ∼ 40 days (see, ref. [38]) is reached for Polish market already for time-lags about ten times shorter. As a result the distribution of returns for time-lags Δ*t* ≥ 1 days starts in case of Polish stocks from the lower value, i.e., *q* = 1.2 ÷ 1.3 in comparison with the one (*q* = 1.4) reported for the mature US market in ref. [38].

A much deeper description of memory effects in stock data requires investigation of the asymmetry in probability distribution of returns. The standard way to measure such asymmetry is based on calculation of skewness defined as the third moment of normalized and centered return distribution:

$$\begin{array}{c} A\left(\Delta t\right)={\left\langle r^{3}\left(t,\Delta t\right)\right\rangle }_{T} \end{array}$$ (10)

with the average taken over considered time window of length *T*. However, following this definition, the main influence on skewness is made by asymmetry of most frequent events accumulated around the head of distribution. As was already mentioned, we want to focus in this paper on eventual difference in distribution shape of whole data, influencing however the behavior of distribution tails. Therefore, we propose to register the presence of such asymmetry quantitatively from the fitted values of *q*^+^ and *q*^−^ parameters describing more precisely the shape of distribution tails. The latter approach will also make possible to introduce the *relative* measure of the whole distribution and simultaneously its asymptotic behavior for large |*r*| instead absolute measure of tails asymmetry. The gain/loss asymmetry of distribution tails is known in financial literature, where the leverage effect [39]–[41] is quoted to have an impact on observable asymmetry [42]. It was also a subject of study in econophysics (see, e.g., [43]–[45]) with the first observation of gain/loss asymmetry in terms od Tsallis distribution in [34]. We will not focus in this paper on detailed microscopic descriptions of phenomena that may stay behind the observable macroscopic effects. Instead we will concentrate on the link between macroscopic description of complex phenomena in financial systems (in terms of non-extensive statistics of available data) and the *a priori* known macroscopic state of the considered financial stock markets. However, relations with microscopic phenomena may be postulated and targeted in this context as the promising further step of deeper analysis.

**Fig 8:**
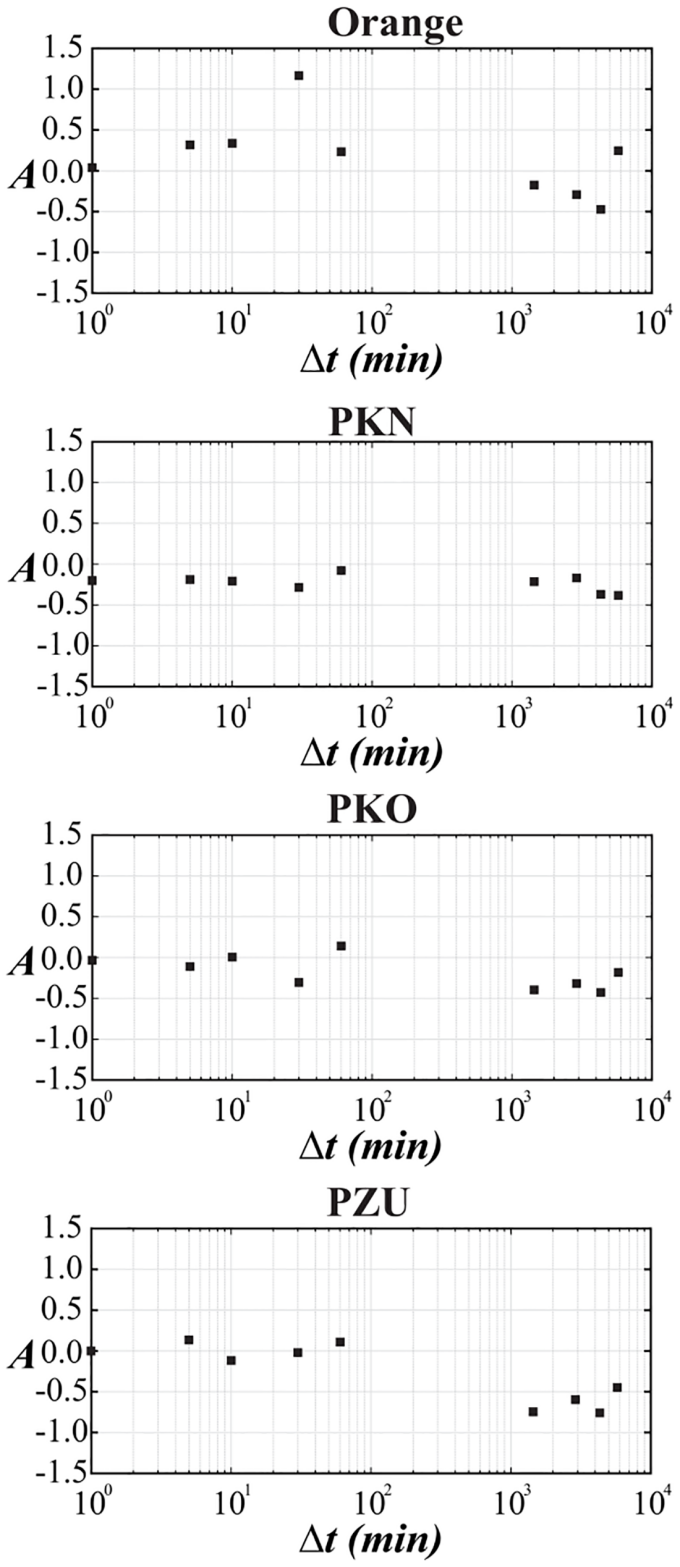
Dependence between skewness *A* versus the time-lag Δ*t* in log-linear scale for chosen companies from WIG 30 stock index. The results seem to be much less informative than those from Fig 6.

The corresponding results of skewness of distributions from Figs 1–5 are collected for comparison with non-extensive formulation of the problem in Table 3. They are also shown in the bottom panel of Fig 7 and in Fig 8 and seem to be much less informative than information coming from Fig 6. We observe that the main Tsallis index *q* decreases with growing Δ*t* for all stock and WIG 30 data and reaches the asymptotic value close to *q* ≃ 1.2 at Δt≳1 day. The asymmetry index defined as *δq* ≡ *q*^+^ − *q*^−^ does not show any uniform behavior along Δ*t*. The same applies to skewness (see Fig 8) where it is difficult to provide any regular functional dependence between skewness and time lags for particular stocks. However, the use of *q*^±^ allows to discuss the relative asymmetry of tails with respect to the level of memory contained in a signal what will be discussed below.

**Table 3:** Skewness of price returns for chosen stokcs from WIG 30 stock index.

Δ*t*	OrangePL	PKN Orlen	PKO BP	PZU	WIG 30
1 min	0.041	-0.200	-0.030	0.001	-0.186
5 min	0.319	-0.189	-0.112	0.138	-0.205
10 min	0.336	-0.208	0.005	-0.114	-0.092
30 min	1.164	-0.285	-0.303	-0.018	-0.558
60 min	0.232	-0.079	0.142	0.110	-0.465
1 day	-0.175	-0.212	-0.398	-0.745	-0.172
2 days	-0.292	-0.169	-0.318	-0.596	0.195
3 days	-0.473	-0.372	-0.431	-0.755	0.048
4 days	0.244	-0.381	-0.179	-0.448	-0.129

Note that positive and negative returns behave in details in different way what is particularly evident from right panels of Fig 6 showing the detailed characteristics of the left and right distribution tails. Usually the relation *q*^−^ > *q*^+^ holds while only *q*^+^ achieves often *q*^+^ ∼ 1 in interday regime. Thus positive returns become faster random (the random case corresponding to Gaussian distribution reflects the limit *q*^+^ = 1) while the memory in negative returns is still kept for interday data. This acknowledges the longer memory existing for the price declines than for price increases. In other words the bad events are remembered longer on stock market than good events. The asymptotic values of *q*^+^ for some stocks (Orange, PZU) reach slightly higher value *q* ∼ 1.2 ÷ 1.3 in interday regime Δ*t* ≤ 4 days—already mentioned as the characteristic value of Tsallis parameter for developed markets.

It is interesting that new information can be extracted if one looks at the *relative* absolute asymmetry between fat tails of positive and negative returns. This relative measure can be defined as follows:

$$\begin{array}{c} \frac{\left|\delta q\right|}{q}=\frac{|q^{+}-q^{-}|}{q} \end{array}$$ (11)

The relative asymmetry index |*δq*|/*q* we introduce here does not have any corresponding quantity (counterpart) in terms of skewness of probability distribution. It can be interpreted as a new quantifier measuring the rescaled asymmetry between temptation among investors (letting the stock to go up) and the level of fear among traders (pushing the stock down). This rescaling is made with respect to so to say “an average level of memory” in data reflected by value of *q* (*q* → 1 corresponds to lack of memory in data). One may expect that the relative asymmetry of tails should also be less noticeable for the whole market index (the capital-weighted average measure of stock prices) than for returns of most particular stock. The corresponding values of this index for stocks from WIG 30 are shown in Table 4 and seem to confirm the above statement for both intraday and interday quotations. It is the case particularly regarding the averages calculated over various time-lags.

**Table 4:** Relative asymmetry ratio $\frac{\left|\delta q\right|}{q}$ of Tsallis distribution to stock data on Polish stock market. The data come from best fit results shown in Table 1. The top part of the table is related to intraday data while bottom part contains interday data. The averaged relative asymmetry ratio ${\langle \frac{\left|\delta q\right|}{q}\rangle }_{intra}$ and ${\langle \frac{\left|\delta q\right|}{q}\rangle }_{inter}$ is taken respectively over all intraday time-lags (from 1 min to 60 min) and interday time-lags (from 1 day to 4 days) for individual stocks and the whole WIG 30 index.

	Δ*t*	OrangePL	PKN Orlen	PKO BP	PZU	WIG 30
$\frac{\left|\delta q\right|}{q}$	1 min	4.8%	6.2%	5.7%	0.7%	2.0%
	5 min	0.7%	2.1%	4.9%	1.4%	1.4%
	10 min	2.0%	0.0%	3.4%	1.4%	3.5%
	30 min	6.1%	4.3%	2.2%	0.7%	2.1%
	60 min	1.4%	2.1%	7.0%	3.6%	0.0%
${\langle \frac{\left|\delta q\right|}{q}\rangle }_{intraday}$		3.0%	3.0%	4.6%	1.5%	1.8%
$\frac{\left|\delta q\right|}{q}$	1 day	3.7%	15.2%	1.6%	20.0%	3.4%
	2 days	12.9%	9.2%	12.9%	26.3%	8.3%
	3 days	13.4%	18.3%	11.9%	26.3%	6.4%
	4 days	13.6%	20.9%	24.2%	13.2%	15.0%
${\langle \frac{\left|\delta q\right|}{q}\rangle }_{interday}$		10.9%	15.9%	12.7%	21.5%	8.3%

One finds from Table 4 also some further noticeable regularity. The relative asymmetry index shows much different values between interday and intraday trading. Indeed, the average 〈|*δq*|/*q*〉 value of |*δq*|/*q* taken over various time-lags is found ${\langle |\delta q|/q\rangle }_{intra}≲5\%$ while the corresponding average value for interday returns reads ${\langle |\delta q|/q\rangle }_{inter}≳10\%$ for most of stocks. Note that due to accuracy of fitted *q*^±^ and *q* values (∼5 × 10^−3^) the existence of relative absolute asymmetry |*δq*|/*q* given by Eq (11) is justified only if |δq|/q≳0.7%. Therefore, its existence in tails of return distribution can be well confirmed here for both: intra- and interday data. Moreover, the average relative asymmetry index is about 3 ÷ 15 times larger (depending on particular stock) for interday returns than for intraday data. We hope to have an explanation of this phenomenon. It is likely that the mechanism responsible for the observed duality relies on different methods basically used by investors for intraday and interday trading. In the first case trading is done mostly by institutional investors who trade usually in shorter time scale using sophisticated stochastic econometric models and numerical applications of high frequency trading (HFT) to predict the behavior of market in short time horizon. In case of interday trading other type of investors—individual traders—are involved who use more traditional technical analysis in a longer time horizon. This lead to interesting observation that trading in longer time horizon involves more asymmetric price speculation, i.e., on the average it has features of less balanced temptation over fears among investors.

**Fig 9:**
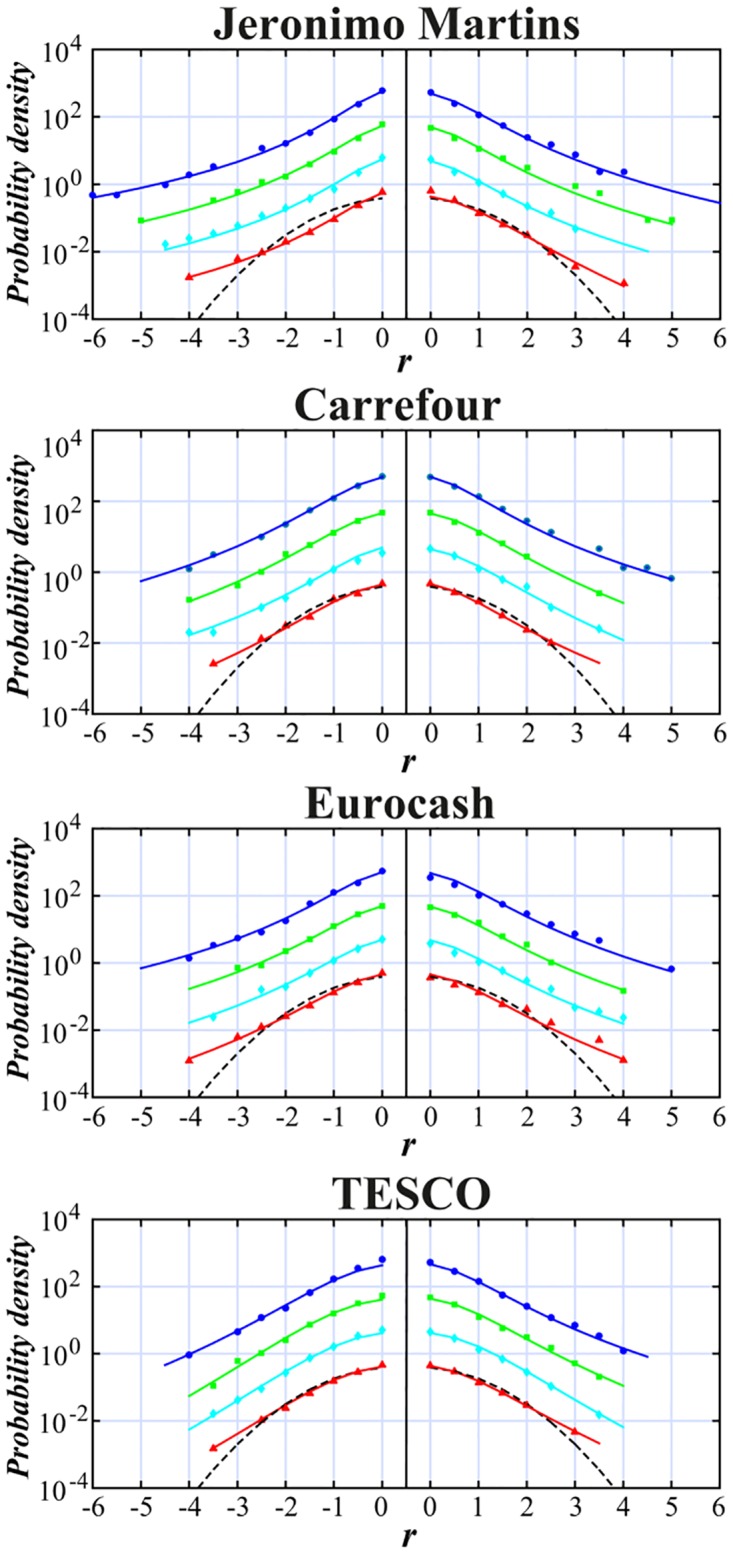
Probability distribution (blue point—1 day, green square—2 days, cyan diamond—3 days, red triangle—4 days) and *q*-normal distribution fit (solid lines) to normalized and centered returns of the leading European food-industrial stores. Interday data are collected from the period July 2005—July 2015 for time-lag 1 ÷ 4 days. All plots are shifted vertically in a similar manner as in Figs 1–5 and compared with normal distribution (dashed line). Shown are results of independent fit to positive and negative returns.

Let us now see how this kind of analysis may help in comparative study of stocks from the same or very similar economic sectors, however listed on different European stock markets—with diversified maturity level. Our study based on the analysis of Tsallis parameters should allow to point out how the shape of fat tails of distribution and their asymmetry may be linked to maturity of the market and to the level of speculative transactions involved in trading. We considered for this purpose four similar large European companies managing the food-industrial network trade. They sell their products in the whole Europe however, they are listed on different European stock markets. Our choice was as follows: Eurocash listed on Warsaw Stock Exchange (WSE), Jeronimo Martins (the owner of large “Biedronka”(Ladybug) supermarket network in Poland) traded on Euronext Lisbon, Carrefour traded on Paris Stock Exchange (PAR) and TESCO traded on London Stock Exchange (data taken from: http://stooq.pl/). The goal was to examine if these companies do share similar features of fat tail distribution of returns for the comparable time-lags and, in particular, the similar asymmetry level between positive and negative returns of their stocks. These outcomes are illustrated in Fig 9 with independent fit made to both tails of probability distribution. The results are also collected quantitatively in Table 4. We investigated in this case only interday data due to the absence of reliable intraday quotations for separate foreign stocks. Nevertheless, even for interday quotations some interesting observations can be done.

One may easy notice that the main Tsallis parameter value *q*, calculated with two significant decimal digits, decreases with Δ*t* for all mentioned stocks in a similar manner as it decreases for stocks within WIG 30 index. The only exception is made by Jeronimo Martins. The latter one seems to keep *q* ∼ 1.5 (see Table 5) for all interday data up to one trading week. This is very exceptional among other developing stocks discussed previously on WSE and also unusual comparing with results published so far for developed markets in [38]. It can also be noticed that the relative asymmetry indicator |*δq*|/*q* is much larger for Jeronimo Martins than for other stocks of the same sector in EU. Its monotonically increasing value with Δ*t* starting from the time lag Δ*t* = 2 days is similar to most companies quoted on the Polish stock market but opposite to the behavior of other stocks of the same sector in Europe. Overall, the average 〈|*δq*|/*q*〉_*inter*_ value for Jeronimo is several times higher than for corresponding companies from other mature European markets. Simultaneously, it is similar to the relative asymmetry for stocks of various sectors on the younger Polish market. In other words the new tail asymmetry indicator shows that the trading features (particularly connected with larger absolute returns) can be more dependent on maturity level of the stock market on which the particular company is quoted than on the economic sector to which this company actually belongs. Indeed, note that these markets were built in different time (Euronext Lisbon—established 2002, WIG (Warsaw)- established 1991, CAC40 (Paris)—established 1987, FTSE (London)—established 1984).

**Table 5:** Difference of Tsallis parameters *δq* = *q*^+^ − *q*^−^, the main Tsallis parameter value *q*, the relative asymmetry ratio |*δq*|/*q* and the averaged relative asymmetry ratio 〈|*δq*|/*q*〉 for the food-industrial trading companies quoted on different European stock markets but making the same economic sector.

Δ*t*	Tsallis index	Jeronimo	Carrefour	Eurocash	TESCO
1 day	*δq*	-0.11	0.03	-0.06	0.10
	*q*	1.47	1.39	1.38	1.28
	|*δq*|/*q*	7.5%	2.2%	4.3%	7.8%
2 days	*δq*	-0.07	-0.03	-0.04	0.10
	*q*	1.45	1.34	1.37	1.20
	|*δq*|/*q*	4.8%	2.2%	2.9%	8.3%
3 days	*δq*	-0.08	-0.11	-0.03	0.12
	*q*	1.46	1.37	1.38	1.18
	|*δq*|/*q*	5.5%	8.0%	2.2%	1.7%
4 days	*δq*	-0.25	0.05	-0.01	0.08
	*q*	1.48	1.31	1.32	1.20
	|*δq*|/*q*	16.9%	3.8%	0.8%	6.7%
〈|*δq*|/*q*〉		8.7%	4.1%	2.5%	6.1%

It is confirmed in literature that the memory effects (and speculation inseparably connected with them and regarded as their consequence) are much stronger for younger markets than for established ones and change also with institutional modifications on the market and market microstructure which is different in mature and emerging markets and may stand behind this phenomena [46]–[48]. These findings may support then the hypothesis that the newly introduced quantifier of asymmetry level in tails of return distributions can be used for detection of speculation in trading. Note that the significant asymmetry between tails of return distribution may exist independently on the *q* value—both in a case of inverse cubic law as well as for the asymptotic limit *q* ∼ 1.2 of mature markets (see Table 5). It is also the case of stocks forming WIG 30 index (see Tables 1 and 4). The role of this asymmetry seems to be underestimated so far in the existence and detection of different memory effects in positive and negative returns. The introduced asymmetry indicator can be postulated as an additional tool for checking the presence of memory in data. The existence of such memory in signal does not seem to be fully verified with the use of one Tsallis main average index value *q* alone. The independent *q* values for separate positive and negative returns make the source of new important information on the financial system.

**Fig 10:**
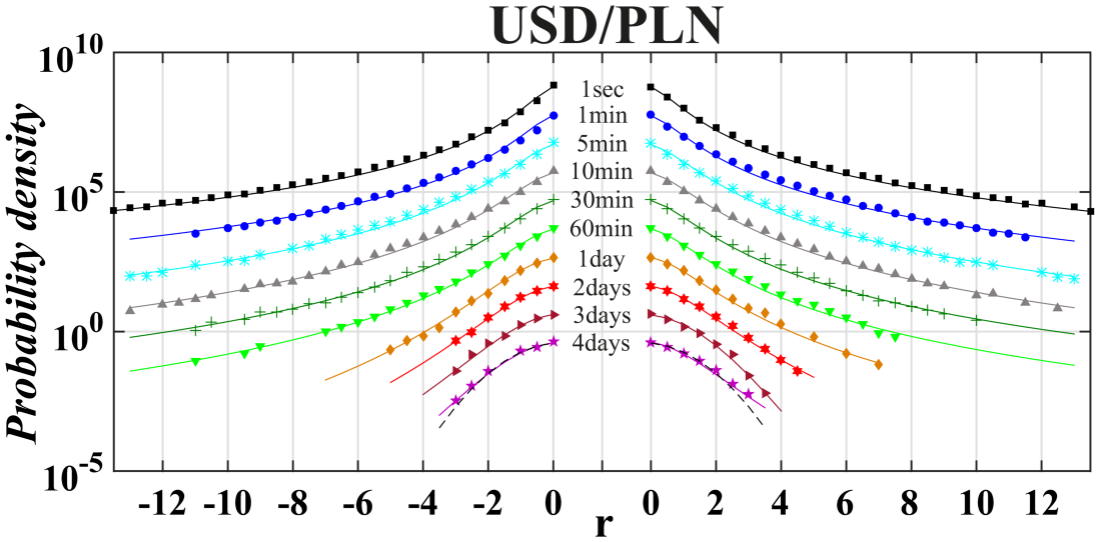
Probability density function in log-linear scale of normalized and centered returns for USD/PLN exchange rates calculated for various time-lags Δ*t* from the period: January 2013—December 2015. The plots for different Δ*t* are drawn as dots and are relatively vertically shifted for better display similarly as Figs 1–5. The dashed line corresponds to Gaussian distribution while solid lines present the best fit of *q*-normal Tsallis distribution separately fitted to positive and negative returns.

**Fig 11:**
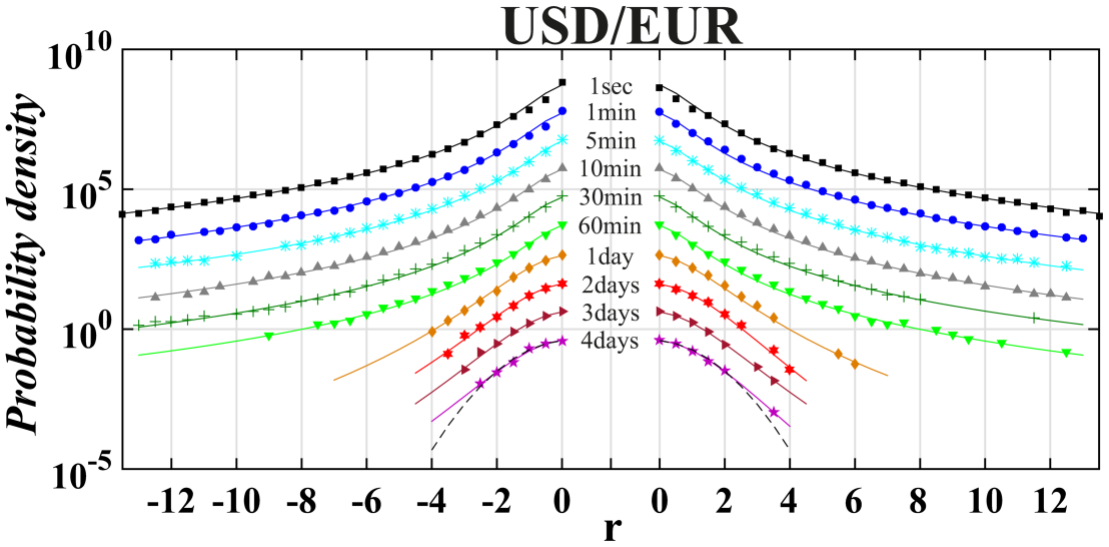
Same as in Fig 10 for USD/EUR exchange rate.

**Fig 12:**
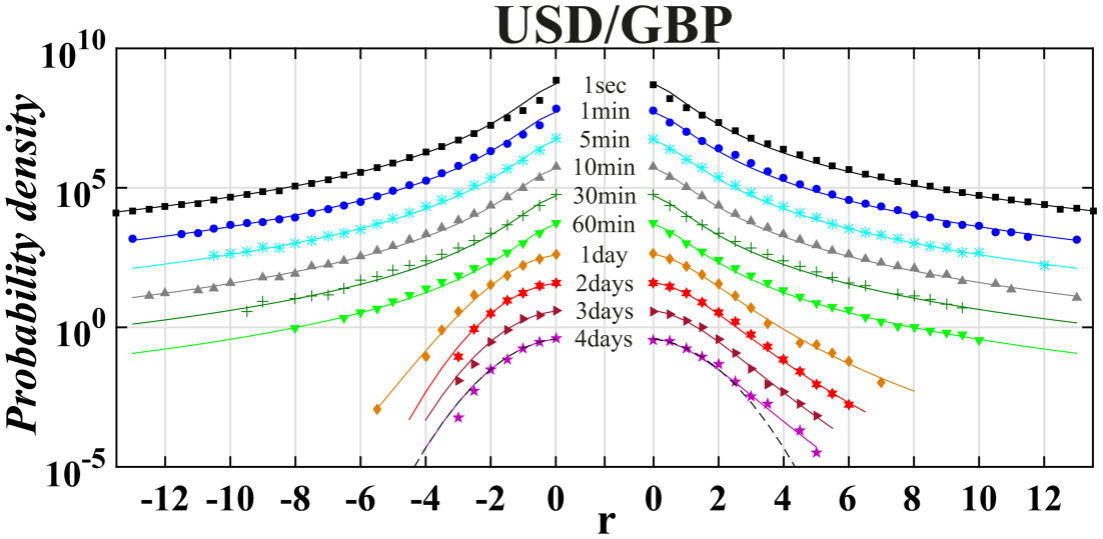
Same as in Fig 10 for USD/GBP exchange rate.

**Fig 13:**
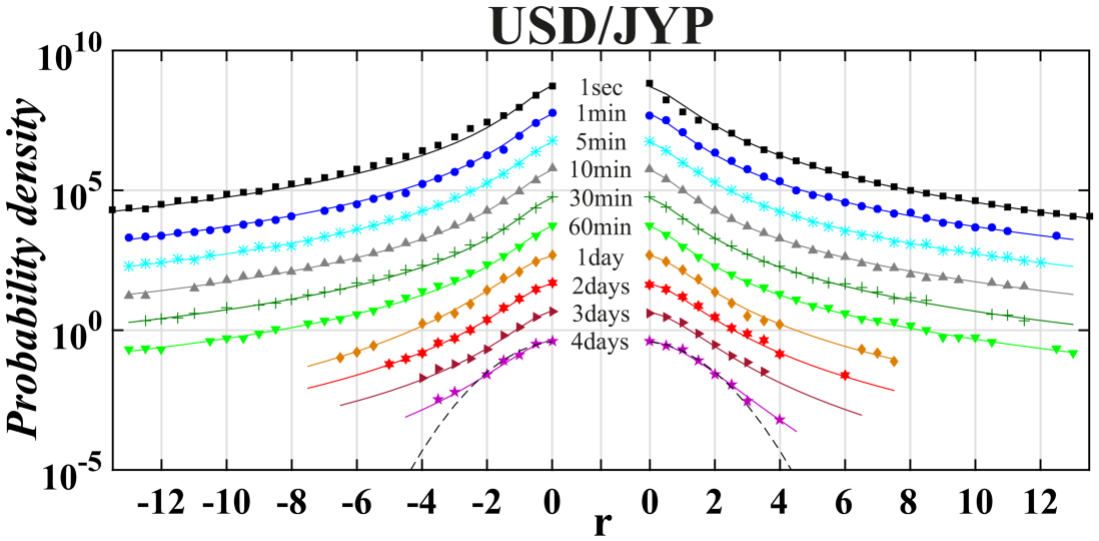
Same as in Fig 10 for USD/JYP exchange rate.

**Fig 14:**
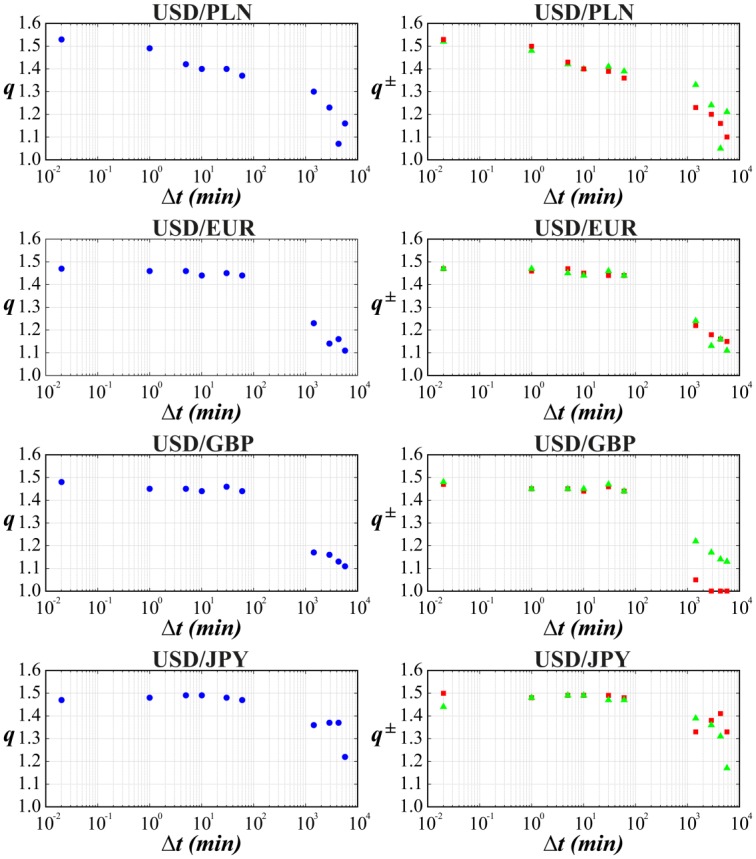
Dependence between Tsallis parameters. *q* (blue point—all returns) and *q*^±^ (green triangle—positive returns, red square—negative returns) (found from Figs 10–13 and collected in Table 7) versus the time-lag used for calculation of returns.

Finally, we applied a tool of non-extensive statistical physics to detect the asymmetry level in distribution of returns on the world most liquid financial market, i.e., money market. This analysis took into account the returns of exchange rates of the most traded currencies on Forex, i.e., USD, EUR, GBP and JYP (data taken from: http://www.histdata.com/). The Polish złoty (PLN) had also been added by us to this money basket because the Polish currency is a good example of the leading currency of the biggest developing European country outside Euro-zone. All results previously obtained for stock data have been shown in a similar manner in Tables 6 and 7. The Table 6 collects outcomes of the best fit of the symmetric or asymmetric *q*-normal distributions to distributions of interday and intraday returns USD/EUR, USD/GBP, USD/JYP and USD/PLN in the period January 1, 2013—December 31, 2015. The quality of fit is supported by consecutive Figs 10–13. For better visualization the Tsallis indices *q* and *q*^±^ have been indicated in Fig 14 as a function of time-lag Δ*t* in a similar manner as shown previously in Fig 6 for separate stocks.

**Table 6:** Results of *q*-normal distribution fit to statistics of returns for main exchange rates on Forex. Polish currency (PLN—złoty) has been added for comparison. Shown are also results of independent fit of Tsallis parameter *q*^±^ to the right (positive returns) and left (negative returns) tail of probability distribution for diversified time-lags.

Δ*t*	Tsallis index	USD/PLN	USD/EUR	USD/GBP	USD/JYP
1 sec	*q*^−^	1.53	1.47	1.47	1.50
	*q*	1.53	1.47	1.48	1.47
	*q*^+^	1.52	1.47	1.48	1.44
1 min	*q*^−^	1.50	1.46	1.45	1.48
	*q*	1.49	1.46	1.45	1.48
	*q*^+^	1.48	1.47	1.45	1.48
5 min	*q*^−^	1.43	1.47	1.45	1.49
	*q*	1.42	1.46	1.45	1.49
	*q*^+^	1.42	1.45	1.45	1.49
10 min	*q*^−^	1.40	1.45	1.44	1.49
	*q*	1.40	1.44	1.44	1.49
	*q*^+^	1.40	1.44	1.45	1.49
30 min	*q*^−^	1.39	1.44	1.46	1.49
	*q*	1.40	1.45	1.46	1.48
	*q*^+^	1.41	1.46	1.47	1.47
60 min	*q*^−^	1.36	1.44	1.44	1.48
	*q*	1.37	1.44	1.44	1.47
	*q*^+^	1.39	1.44	1.44	1.47
1 day	*q*^−^	1.23	1.22	1.05	1.33
	*q*	1.30	1.23	1.17	1.36
	*q*^+^	1.33	1.24	1.22	1.39
2 days	*q*^−^	1.20	1.18	1.00	1.38
	*q*	1.23	1.14	1.16	1.37
	*q*^+^	1.24	1.13	1.17	1.36
3 days	*q*^−^	1.16	1.16	1.00	1.41
	*q*	1.07	1.16	1.13	1.37
	*q*^+^	1.05	1.16	1.14	1.31
4 days	*q*^−^	1.10	1.15	1.00	1.33
	*q*	1.16	1.11	1.11	1.22
	*q*^+^	1.21	1.11	1.13	1.17

**Table 7:** Relative asymmetry ratio $\frac{\left|\delta q\right|}{q}$ of Tsallis distribution to return data on Forex market for chosen main exchange rates and Polish złoty (PLN). Input data are taken from Table 6. The top part of the table is related to intraday returns while the bottom part with interday returns in a similar manner as in Table 4.

	Δ*t*	USD/PLN	USD/EUR	USD/GBP	USD/JYP
$\frac{\left|\delta q\right|}{q}$	1 sec	0.7%	0.0%	0.7%	4.1%
	1 min	1.3%	0.7%	0.0%	0.0%
	5 min	0.7%	1.4%	0.0%	0.0%
	10 min	0.0%	0.7%	0.7%	0.0%
	30 min	1.4%	1.4%	0.7%	1.4%
	60 min	2.2%	0.0%	0.0%	0.7%
${\langle \frac{\left|\delta q\right|}{q}\rangle }_{intra}$		1.1%	0.7%	0.3%	1.0%
$\frac{\left|\delta q\right|}{q}$	1 day	7.7%	1.6%	14.5%	4.4%
	2 days	3.3%	4.4%	14.7%	1.5%
	3 days	10.3%	0.0%	12.4%	7.3%
	4 days	9.5%	3.6%	11.7%	13.1%
${\langle \frac{\left|\delta q\right|}{q}\rangle }_{inter}$		7.7%	2.4%	13.3%	6.6%

The same noticeable difference between relative asymmetry ratio for interday and intraday returns of exchange rates can be observed here (〈|*δq*|/*q*〉_*inter*_ = 0.3% ÷ 1.1% for Δ*t* = 1 ÷ 3600 sec and 〈|*δq*|/*q*〉_*intra*_ = 2.4% ÷ 13.3% for Δ*t* = 1 ÷ 4 days, depending on the currency pair) as for stock markets. This indicates probable different strategies applied by investors in a short and longer time horizon. We discover also, that the relative asymmetry ratio |*δq*|/*q* shown in top part of Table 7 for intraday time-lags is much smaller than in case of stock data. It lies almost completely within confidence level of asymmetry absence between tails (|*δq*|/*q* < 1%). Therefore, the asymmetry of distribution tails in case of Forex can be confirmed only for interday data.

It is also worth noting that on average |*δq*|/*q* is noticeably higher for interday USD/GPB exchange rates (comparing with other discussed currency quotations) and TESCO stock (comparing with other stocks of the same economic sector in UE)—see Tables 5 and 7. This might be an independent signal of upcoming problems with UK membership in the European Union seen already before 2014. In fact, strong suggestions for very likely referendum on the stay of Great Britain in EU and the possible Brexit had already taken place in years 2013—2014 (see, e.g., http://www.economist.com/blogs/graphicdetail/2016/02/graphics-britain-s-referendum-eu-membership). These circumstances should have influenced the state of the market following the growing lack of confidence that UK will surely further stay in EU. The data analyzed here were taken exactly from this period (January 2013—December 2015). Although the above remark may seem to be very preliminary and speculative, one should be aware that investors usually “buy rumors and sell facts” on the market. There are many examples of how the “buy rumors, sell facts” principle affects the foreign exchange market every day. Waiting for the publication of positive (negative) results can produce an optimistic (pessimistic) response not initially directly shown in the actual movement of the price expressed as trend. Only following the publication of the report, the difference between the information contained therein and the market consensus is verified by investors and could eventually lead to a reversal of the current direction of the market. We believe that the asymmetry indicator we introduced here may take the role of one of the “hidden” indicators [49], [50] that can show the actual state of the market much before the critical-like phenomena (crash, rupture point, etc.) do occur.

The second interesting (but not surprising) observation is that the average relative asymmetry 〈|*δq*|/*q*〉 between both tails of daily return distributions for Forex market is closer to asymmetry characteristic for stocks quoted on mature markets than for stocks traded on emerging markets (compare with data in Table 4).

## Concluding remarks

In this paper we used the methods of non-extensive statistical physics to describe quantitatively the statistics of returns on stock and money markets. We provided also the interpretation how parameters of complex financial systems evaluated within the approach based on non-extensive statistical physics can be used to describe the current state of the market and in particular the balance between fear to loose and the temptation to earn—the main engine of any trading. In this context a particular caution was given to the asymmetric behavior of investors reflected in the asymmetry between tails of normalized and centered returns. The new method of measuring this asymmetry has been proposed which is constructed on Tsallis non-extensive *q* parameters monitored separately for distribution of positive and negative returns. This fills an existing gap in literature about the meaning, interpretation and behavior of the non-extensive Tsallis parameter *q*. A comparison of parameter’s values of non-extensive complex financial systems which are calculated for different markets and the related detailed analysis of individual stocks may lead to information and interpretations useful for investors and traders.

We were able to observe in the case of the main developing market in Europe—Polish stock market—that the inverse cubic power law (*q* = 3/2) can be very well recognized for WIG 30 stock index for time-lags Δ*t* = 1 min. The same is the case for the most of its constituent stocks for 1 ≤ Δ*t* ≤ 30 min. However, a remarkable departure from the inverse cubic law is observed already for Δ*t* ∼ 60 min (corresponding to *ζ* = 4—see Eq (3)). The statistics of interday returns on WSE starts from the lower value *q* = 1.2 ÷ 1.3 than reported in literature for the established and mature markets. The Polish emerging market turns out to obtain also faster the equilibrium state (at *q* = 1.2) reported elsewhere as characteristic for the mature markets.

An independent fit of Tsallis distribution to positive and negative returns has been proposed by us as the way for effective measure of the asymmetry of fat tails of return distribution. This approach seems to have intriguing practical applications. We argued that information on both *δq* = *q*^+^ − *q*^−^ and *q* values are *simultaneously* necessary to get information about the state of the complex financial system (i.e., included memory level in series of positive and negative returns, hence, the level of speculations, etc.) The new quantifier |*δq*|/*q* of the relative asymmetry between distribution tails has been introduced therefore. Its properties were investigated for variety of stocks and time-lags. The behavior of |*δq*|/*q* values turns out to serve much more detailed description of data than the standard skewness measure does. Moreover, the standard skewness measures the absolute asymmetry between distribution tails and contrary to new quantifier does not allow to make the relative characteristics with respect to the level of memory included already in data.

From the variety of data connected in intraday and interday trading we concluded usefulness of the newly introduced relative asymmetry quantifier for independent detection of speculation level on a stock market and we argued about asymmetric memory effect between positive and negative returns, or more generally about longer memory existing for price declines than for price increases. We made a trial to explain the mechanism of such asymmetry linking it to different investors strategies applied in a shorter and longer time horizon. We observed also that positive returns tend to become random (*q*^+^ → 1) usually much faster than negative returns do. This asymmetry strongly depends on the frequency of data sampling and the trials to describe the left and the right tail of return distribution with just one value of *q* makes a clear simplification of the problem. This idea was then discussed on examples of mature as well as for growing markets like Polish stock market and has been applied also for the most liquid markets like Forex money exchange market.

It has been widely confirmed within this paper that that value of |*δq*|/*q* is remarkably higher for younger markets than for well established ones. Thus the value of this quantifier reflects the presence of more unbalanced emotions among traders. This may be the signature of the speculation level involved in trading. The relative asymmetry was also checked to be less remarkable for the whole market index than for returns of particular stock. Our analysis confirmed that the high and low frequency market data make two different worlds with different level of memories contained in them. However, in case of money market, the high frequency data practically do not show the meaningful relative asymmetry ratio |*δq*|/*q*. Such behavior of Forex is opposite to stock data. The sensitivity of the new asymmetry parameter was confirmed by its slightly higher values for TESCO stocks and USD/GBP exchange rate returns. In our opinion, this can be a result of investors’ concern about British market and the possibility of Brexit (already visible in the years 2013—2015)—finally concluded in the referendum of 2016.

The proposed asymmetry measure based on non-extensive statistical properties of stock market was also used to study similarities and differences between stocks from the same economic sector however, traded on stock markets of diverse maturity level. We have chosen examples of food-industrial supermarket owners for this purpose. We identified that the relative asymmetry indicator 〈|*δq*|/*q*〉_*inter*_ is much larger for Jeronimo-Martens listed on the youngest Euronext Lisbon—Portugal stock market than for other stocks (except TESCO) and grows with time lag Δ*t* similarly to most companies quoted on the Polish market in Warsaw. This is contrary to behavior of other stocks of the same sector in Europe. Hence, it may indicate more intensive speculative trading within the relatively youngest stock market in UE.

We conclude that the new asymmetry quantifier |*δq*|/*q* seems to be a very precise tool of investigation of trading market features and should be checked further in details in a view for other applications.
